# Altered KLOTHO and NF-κB-TNF-α Signaling Are Correlated with Nephrectomy-Induced Cognitive Impairment in Rats

**DOI:** 10.1371/journal.pone.0125271

**Published:** 2015-05-11

**Authors:** Sabrina Degaspari, Carmen Branco Tzanno-Martins, Clarice Kazue Fujihara, Roberto Zatz, João Paulo Branco-Martins, Tania Araujo Viel, Hudson de Souza Buck, Ana Maria Marques Orellana, Ana Elisa Böhmer, Larissa de Sá Lima, Diana Zukas Andreotti, Carolina Demarchi Munhoz, Cristoforo Scavone, Elisa Mitiko Kawamoto

**Affiliations:** 1 Molecular Neuropharmacology Laboratory, Department of Pharmacology, Institute of Biomedical Science, University of São Paulo, São Paulo, Brazil; 2 Centro Integrado de Nefrologia, São Paulo, Brazil; 3 Renal Division, Department of Clinical Medicine, Faculdade de Medicina, Universidade de São Paulo, São Paulo, Brazil; 4 School of Arts, Sciences and Humanities, Universidade de São Paulo, São Paulo, Brazil; 5 Department of Physiological Sciences, Santa Casa de São Paulo Medical School, São Paulo 01221–020, Brazil; The University of Manchester, UNITED KINGDOM

## Abstract

Renal insufficiency can have a negative impact on cognitive function. Neuroinflammation and changes in klotho levels associate with chronic kidney disease (CKD) and may play a role in the development of cognitive impairment (CI). The present study evaluates the correlation of cognitive deficits with neuroinflammation and soluble KLOTHO in the cerebral spinal fluid (CSF) and brain tissue of nephrectomized rats (Nx), with 5/6 renal mass ablation. Nx and sham Munich Wistar rats were tested over 4 months for locomotor activity, as well as inhibitory avoidance or novel object recognition, which started 30 days after the surgery. EMSA for Nuclear factor-κB and MILLIPLEXMAP or ELISA kit were used to evaluate cytokines, glucocorticoid and KLOTHO levels. Nx animals that showed a loss in aversive-related memory and attention were included in the CI group (Nx-CI) (n=14) and compared to animals with intact learning (Nx-M n=12 and Sham n=20 groups). CSF and tissue samples were collected 24 hours after the last behavioral test. The results show that the Nx-groups have increased NF-κB binding activity and tumor necrosis factor-alpha (TNF-α) levels in the hippocampus and frontal cortex, with these changes more pronounced in the Nx-CI group frontal cortex. In addition, the Nx-CI group showed significantly increased CSF glucocorticoid levels and TNF-α /IL-10 ratio compared to the Sham group. Klotho levels were decreased in Nx-CI frontal cortex but not in hippocampus, when compared to Nx-M and Sham groups. Overall, these results suggest that neuroinflammation mediated by frontal cortex NF-κB, TNF-α and KLOTHO signaling may contribute to Nx-induced CI in rats.

## Introduction

Chronic kidney disease (CKD) is a public health problem that has been related to cognitive decline in recent years [1, 2]. Cognitive impairment (CI) is linked to low medication adherence, which is a major cause of morbidity in CKD [3]. Although systemic cardiovascular diseases (CVD) is a well-known risk factor for CKD, nontraditional risk factors such as inflammation may be more prominent in individuals with CKD and may also predispose to CVD and cerebrovascular disease [4]. Evidence suggests that renal inflammation, involving cytokines, chemokines and growth factors, is a key factor in the pathogenesis of CKD [5, 6]. Increased systemic inflammation, including increased plasma proinflammatory cytokines and reactive oxygen species (ROS), are found in CKD patients [6]. However, little data exist on how inflammation in CKD may contribute to abnormal brain function.

Inflammation is increased in CKD and may play a role in the development of CI [1, 7]. It is known that cytokines play an important role in the molecular mechanisms underlying memory and cognitive processes [8, 9]. Chronic pro-inflammatory mediators disrupt hippocampal neuronal functions, including long-term potentiation and working memory consolidation [10, 11]. Furthermore, systemic administration of the pro-inflammatory cytokine tumor necrosis factor (TNF-α) reduces cell proliferation in the hippocampus [12]. Anti-inflammatory glucocorticoid (GC) actions include the blunting of proinflammatory genes, such as *TNFα*, thereby increasing expression of anti-inflammatory gene, such as interleukin-10 (IL-10), and, perhaps most importantly, inhibiting the activity of the pro-inflammatory transcription factor, Nuclear factor-kB (NF-κB)[13].

NF-xB activity is driven by the Rel/NF-κB family proteins, which form homo- and heterodimers through the combination of the subunits p65 (or RelA), p50, p52, cREL and RelB. NF-κB can be activated by cytokines such as TNF-α, as well as by reactive oxygen species (ROS)[14, 15]. NF-κB, which is constitutively expressed in the cytoplasm, is inhibited by a family of molecules termed inhibitor κB (IκBs). Inducers of NF-κB act by intracellular signaling pathways that activate the IκB kinases (IKKs), which phosphorylate two specific N-terminal serines of IκBα, resulting in IκBα polyubiquitination and degradation by the 26S protease [16]. When IκB is degraded, NF-κB migrates to the nucleus, modulating the transcription of many neurodegeneration associated genes [17].

Klotho is a gene involved in premature aging syndromes and cell senescence [18, 19]. In the kidney, Klotho is reduced in CKD patients [20]. Klotho encodes a single-pass transmembrane protein whose extracellular domain is secreted. These two forms of Klotho protein exert distinct functions. Membrane Klotho forms a complex with fibroblast growth factor (FGF) receptors and functions as an obligate co-receptor for FGF23 [21]. Secreted Klotho functions as a humoral factor that regulates activity of multiple glycoproteins on the cell surface, including ion channels and growth factor receptors such as insulin/insulin-like growth factor-1 receptors (IGF1) [22, 23].

Recent evidence shows that TNF-α down-regulates KLOTHO expression through an NF-κB-dependent mechanism [24]. Here we provide the first evidence showing that changes in NF-KB activity and TNF-α-KLOTHO signaling play an important role in nephrectomized (Nx) induced CI in a rodent CKD model.

## Material and Methods

### Animals

Two-months-old adult male Munich-Wistar rats, weighing initially between 230 and 260 g were used in this study. Rats were obtained from a local facility at the University of São Paulo, and maintained at 23 ± 1°C, with relative air humidity at 60 ± 5%, under 12-h light-dark cycle with free access to food and water. Nephrectomy was performed after ventral laparotomy under anesthesia with sodium pentobarbital 50 mg/kg IP (n = 28), by removal of the right kidney and ligation of 2 branches of the left renal artery, resulting in the infarction of two thirds of the left kidney. Sham-operated rats (sham) underwent anesthesia and manipulation of the renal pedicles, without removal of renal mass (n = 22). After recovering from anesthesia, the animals were returned to their original cages, which were kept warm during the following 24 hours. All experimental procedures were approved by the Ethical Committee for Animal Research (CEEA) of the Biomedical Sciences Institute of the University of São Paulo.

### Experimental groups

**Fig 1:**
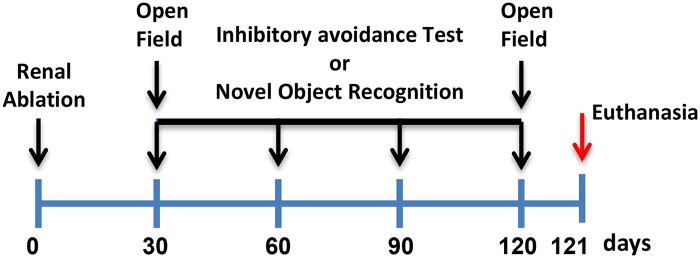
Design of experiments to determine the effects of the 5/6 Nx animal model on cognitive function, NF-kB activity, cytokines levels and KLOTHO levels. For the behavioral experiments, the open field and Inhibitory avoidance behavior or novel object recognition tests were performed thirty days after renal ablation and repeated once a month for more than three months. For the biochemical experiments, 24 hours after the end of the behavioral studies, animals were killed by decapitation with CSF, hippocampal and frontal cortex samples then collected.

Thirty days after renal ablation we started the locomotor activity and Inhibitory avoidance behavior tests or locomotor activity and novel object recognition tests and repeated this monthly (Fig 1). Nx rats formed the experimental groups, which were compared to the sham/control group. These two groups were tested on the behavior learning tasks, with the Nx animals that failed to learn forming the Nx-CI group (n = 15) and compared to experimental animals that acquired learning forming the Nx-M group (n = 13). At the end of this period, animals were killed by decapitation following procedures approved by the CEEA and the CSF, frontal cortex and hippocampus samples were collected. Samples were used to analyze IL-10, Il-1β, IL-6, TNF-α, interferonγ (IFNγ), C-reactive protein (CRP), glucocorticoid (GC) and KLOTHO levels by ELISA test, with NF-kB activity analyzed by electrophoretic mobility shift assays (EMSA).

### Behavioral tests

#### Inhibitory avoidance

The inhibitory avoidance apparatus (Ugo Basile, Comerio, VA, Italy) consists of two acrylic compartments of the same size, one illuminated and the other kept dark, separated by an automatic door. The floors of both chambers were made of stainless steel rods, with the floor of the dark chamber capable of being electrified. During the training session, animals were placed in the lighted compartment and the door was opened. Once the animal entered the dark compartment, the door was closed and an electric foot-shock (0.5 mA, 2 s in duration) was delivered through the grid floor. The animal was removed from the dark compartment and returned to its home cage immediately after receiving this shock. Twenty-four hours later the animals were subjected to the test session. Animals that failed to enter the dark compartment in 5 min were removed from the apparatus and assigned a ceiling score of 300 s. The training session was performed 29 days after Nx surgery. All the animals were retested after 30 days, 60 days and 90 days following the training day. The latency time to cross from the light to the dark side of the chamber was used to determine whether the rats had learned a fear-conditioned stimulus.

#### Novel object recognition

Novel object recognition evaluates memory retention, learning and the preference for novelty as an index of the influence of different brain regions in the process of recognition. This test rewards the innate preference that rats have for selecting novel objects and this preference is displayed by spending more time exploring novel than familiar objects [25]. Object recognition tasks consisted of two phases. In the first phase, the animal was presented with the familiarized object (the sample object) in a distinct environment and freely allowed to explore this object for 10 minutes. Following sample-object exposure, the animal was returned to its home cage and colony for 3 hours forming a retention period. In the second phase, the animal was placed in the environment and exposed to the sample object and to a novel object for 5 minutes. Object recognition was signified by greater exploration time of the novel object.

#### Locomotor activity test

Spontaneous locomotor activity was measured using an electronic animal activity meter (model 7430, Ugo Basile, Comerio, Italy). The apparatus consisted of a transparent acrylic cage (35 cm × 23 cm × 20 cm) with a set of horizontal sensors to register locomotor activity and a set of vertical sensors to register standing activity. Rats were placed in the center of cage and allowed to explore the apparatus for five minutes whilst the horizontal (locomotion) and vertical (rearing) movements were captured. Thereafter, rats were returned to their home cages. The apparatus was cleaned with 5% ethyl alcohol and allowed to dry between tests.

### Biochemical assays

#### Nuclear extract

Nuclear extract of each hippocampus and frontal cortex was prepared as previously described [26]. Briefly, tissues were homogenized using a Dounce homogenizer in cold PBS supplemented with 0.5 mM PMSF, 2.5 μg/ml leupeptin and 2.5 μg/ml antipain, and centrifuged at 4°C for 30 sec at 12,000g. The supernatants (cytoplasmic extract) were reserved for immunoblot, and the pellets were resuspended in lysis buffer (1.5 mM MgCl_2_, 10 mM KCl, 0.1 mM EDTA, 0.5 mM PMSF, 2.5 μg/mL leupeptin, 2.5 μg/ml antipain and 10 mM HEPES; pH 7.9) and incubated on ice for 10 min. After addition of NP-40 (10%), samples were vigorously mixed and centrifuged for 30 sec at 12,000g. Supernatant was discarded, and the pellet was resuspended in extraction buffer (25% glycerol, 1.5 mM MgCl_2_, 300 mM NaCl, 0.25 mM EDTA, 0.5 mM PMSF, 2.5 μg/ml leupeptin, 2.5 μg/ml antipain and 20 mM HEPES; pH 7.9), incubated for 20 min on ice, and centrifuged for 20 min at 12,000xg at 4°C. The resulting supernatants containing nuclear proteins were stored at -80°C. Protein concentration was determined using the Bio-Rad (Quick Start Bradford,Richmond, USA) colorimetric assay [27].

#### EMSA for NF-κB

EMSA for NF-κB was performed by using the gel shift assay kit from Promega (Madison, USA), as described previously [26]. ^32^P-NF-κB double-stranded consensus oligonucleotide probe (5’-AGTTGAGGGGACTTTCCCAGGC-3’; 25,000 cpm) and nuclear extracts (15 μg) were used. DNA—protein complexes were separated by electrophoresis through a 6% nondenaturing acrylamide:bis-acrylamide (37.5:1) gel in 0.53 Tris-borate/EDTA (TBE) for 2 hours at 150 V. Gels were vacuum dried and analyzed by autoradiography. For competition experiments, NF-κB and transcription initiation factor II (TFIID; 5’-GCAGAGCATATAAGGTGAGGTAGGA-3’) unlabeled double-stranded consensus oligonucleotide were included in 20-fold molar excess over the amount of ^32^P-NF-κB probe, in order to detect specific and nonspecific DNA—protein interactions, respectively. Unlabeled oligonucleotides were added to the reaction mixture 20 min before the radioactive probe. Supershift assays, using antibodies against different NF-κB subunits (p50 and p65, 1:20 dilution), were also conducted according to the protocol of the manufacturer (Santa Cruz Biotechnology, Santa Cruz, USA) before the incubation of nuclear extracts with the labeled oligonucleotide [28]. Autoradiographs were visualized with a photo documentation system DP-001-FDC (Vilber Lourmat, Marne la Vallée, France) and quantified in NIH Image J software (Bethesda, USA). Several exposure times were analyzed to ensure the linearity of the band intensities.

#### Tissue TNF-α, CSF Cytokines and GC Levels

To evaluate CNS inflammation we collected the CSF intracisternally after anesthesia, as well as frontal cortex and hippocampus samples. IL-1, TNF-α, IL-6, IL-10, INFγ and CRP were measured using a commercial MILLIPLEXMAP kit (Millipore Corporation, Massachusetts, USA). Tissue TNF-α and CSF GC samples levels were determined by commercial mouse ELISA kit (Enzo Life Science, Exeter, UK).

#### Frontal Cortex and Hippocampus KLOTHO Levels

To measure soluble KLOTHO levels, we used cytoplasmic frontal cortex and hippocampus extracts according to the manufacturer instructions. Briefly, the structures were homogenized in lysis buffer (137 mM NaCl, 20 mM Tris HCl pH 8, 1% NP40, 10% glycerol, 0.5 mM PMSF, 2.5 μg/mL leupeptin, and 2.5 μg/ml antipain), then centrifuged for 30 sec at 12,000g. Supernatant was stored at -80°C. KLOTHO levels were measured by enzyme-linked immunoassay (Uscn Life Science kit, Ontario CANADA).

### Statistical Analyses

Statistical analyses were performed using GraphPad Prism (GraphPad, San Diego, USA). In the inhibitory avoidance task, the heteroscedasticity of data and the use of the 300-second ceiling in test sessions required the use of nonparametric statistics. In this way, data were represented as medians and interquartile range and analyzed using Wilcoxon signed rank test (paired and nonparametric t-test). In order to verify the difference between groups in behavioural tests, Kruskal-Wallis analysis of variance followed by Dunn’s multiple comparison test was performed. Student´s t test were used to compare Nx-M and Nx-CI groups at 120 days after the surgery. All other data were analyzed using a one-way ANOVA followed by Newman-Keuls test. Parametric data were expressed as mean ± SEM, and nonparametric data are given as medians and quartiles. p <0.05 was considered significant.

## Results

### Clinical parameters and histological kidney analyses

Surgical procedures for the induction of CKD did not significantly affect survival. The overall mortality rate was approximately 5% (2 deaths).

Both Nx-M and Nx-CI exhibited a similar pattern of changes in renal function, body weight and blood pressure. However, a significant reduction in body weight was detected in the both Nx groups (Nx-M and Nx-CI) when compared with the sham group after four months (Table 1). The both Nx groups also exhibited an increase in mean systolic blood pressure and in serum creatinine levels compared with the sham group (Table 1).

**Table 1:** Clinical Parameters of CKD in sham and Nx groups after 60 and/or 120 days of the surgical procedure.

	Body Weight (g)		Blood Pressure (mmHg)		Creatinine (mmHg)	Hematocrit(%)
	60 days	120 days	60 days	120 days	120	120
Sham (n = 20)	282.4 ± 4.7	292.5 ± 6.2	102.0 ± 0.3	117.0 ± 0.6	0.66 ± 0.03	46.2 ± 0.6
Nx-M (n = 12)	250.8 ± 5.0*	258.7 ± 4.3*	129.0 ± 1.0*	150.0 ± 1.3*	1.13 ± 0.02*	44.3 ± 0.5
Nx-CI (n = 14)	238.9 ± 4.7*	249.7 ± 2.8*	131.0 ± 0.7*	152.0 ± 1.6*	1.14 ± 0.01*	43.1 ± 0.4

Nx animals that showed a loss in aversive-related memory and attention were included in the CI group (Nx-CI) (n = 14) and compared to animals with intact learning (Nx-M n = 12 and Sham n = 20 groups).

*p < 0.001 versus Sham group—One-way ANOVA followed by Newman-Keuls test. Body Weight: 60 days—F (2,243) = 23.2 and 120 days—F (2,43) = 21.9; Blood Pressure: 60 days—F (2,43) = 716.4 and 120 days—F (2,43) = 326.5; Creatinine—F (2,43) = 416.4.

A significant 14% reduction in body weight was detected in the Nx group compared with the sham group after four months (Table 1). The Nx group also exhibited an increase in mean systolic blood pressure and in serum creatinine levels (approximately 170%) compared with the sham group (Table 1).

**Fig 2:**
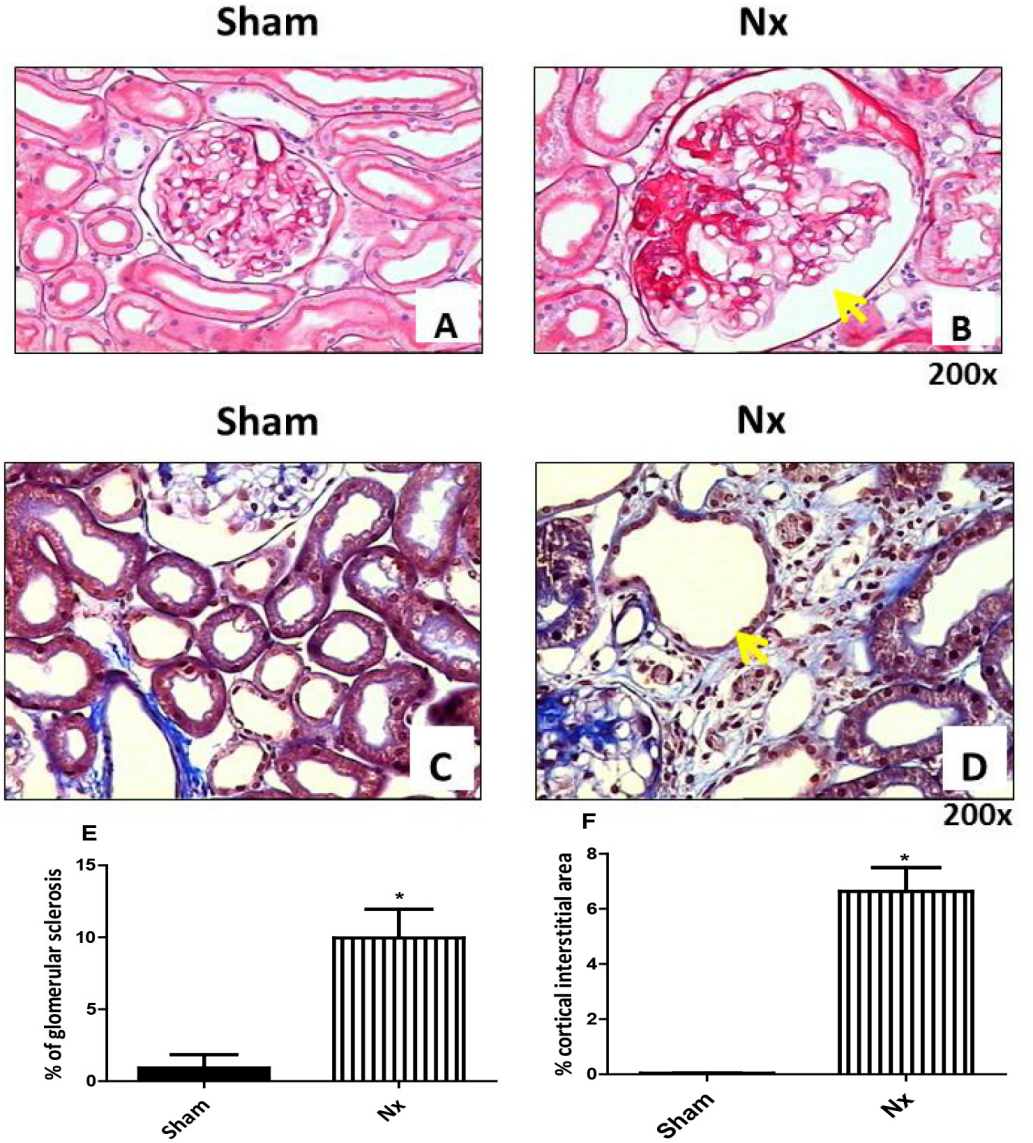
Histological kidney analysis in sham and Nx groups at 121 days after the surgical procedure. (A,B). The photomicrographs are representative samples of glomerular sclerosis observed in sham and Nx kidneys that were stained with periodic acid-Schiff (SPADES). The results showed a significant increase in the number of glomeruli with sclerosis (yellow arrow) in the Nx group (B) (n = 8) when compared to the sham group (A) (n = 5). (C,D) The photomicrographs are representative samples of cortical interstitial area expansion observed in Nx (D) (yellow arrow) when compared to sham (C) that were stained with SPADES. The analysis showed a significant increase in cortical interstitial area in the Nx group (n = 8) when compared to the sham group (n = 5). (E) The graphical representation of the percent of glomerular sclerosis (A,B) and (F) of cortical interstitial area (C,D), respectively. *p<0.05 versus sham—Student's t test.

Histological analysis revealed a significant increase in the percentage of sclerotic glomeruli and an expansion of the cortical interstitial area in the Nx group when compared to the sham group (Fig 2A–2F). These morphological changes were used to confirm that the nephrectomy surgeries were all exactly the same.

### Nx group exhibited CI and attentional deficits

**Fig 3:**
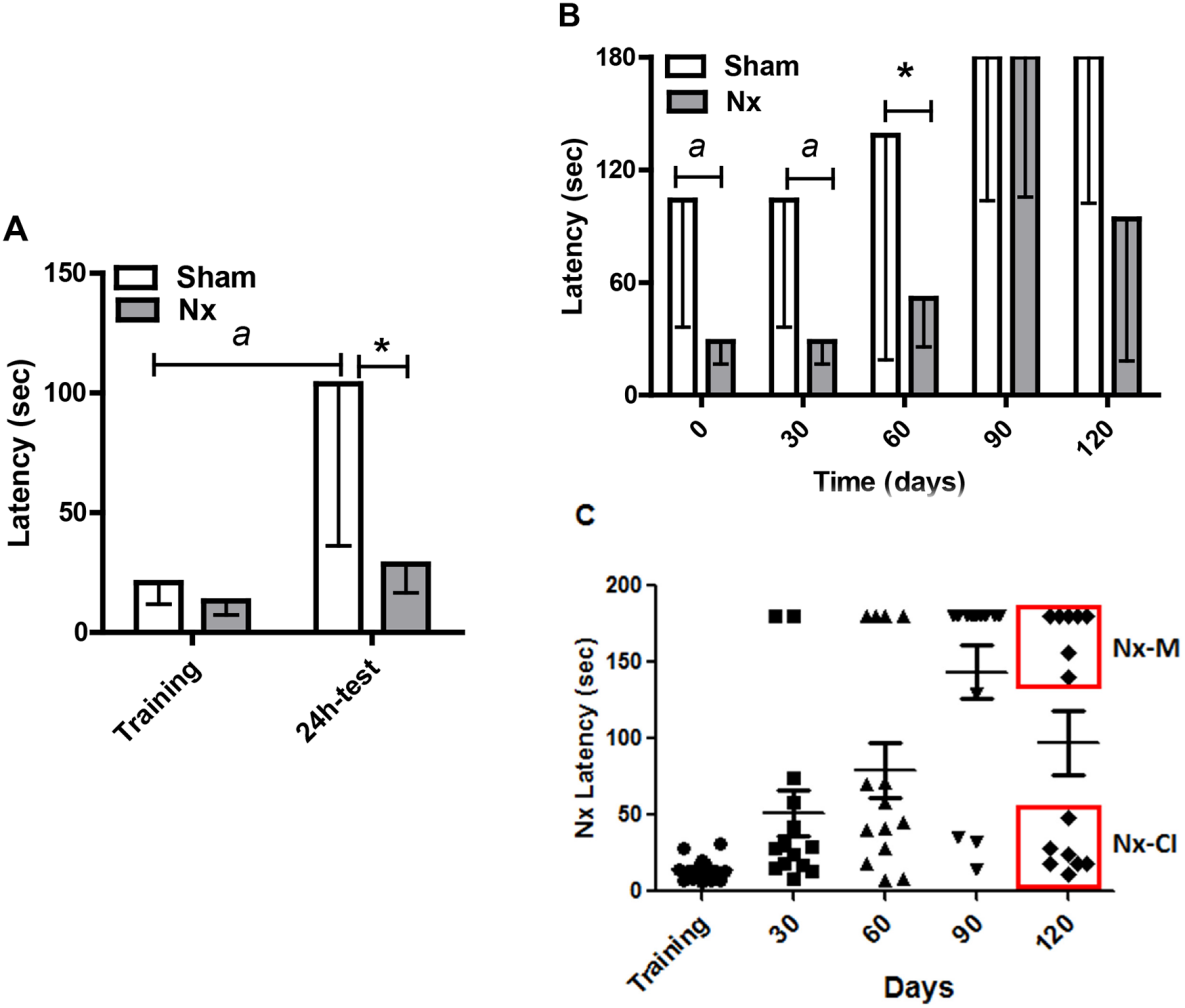
Inhibitory avoidance test analysis in sham and Nx. (A) The first exposure of animals to the inhibitory avoidance equipment and the test performed 24h after the training session. Sham and Nx groups entered the dark space in short time, showing no difference concerning the preference for this ambient but a significant difference between both groups behavior was observed in the test performed 24h after the training session. (B) All the animals were tested at 30, 60, 90 and 120 days following the 24h-test day. During the observational time, 24h-test was considered the 0 (zero) time. The latency time to cross from the light to the dark side of the chamber was measured and used as a reference to distinguish if the rats learned from a fear conditional stimulus. Animals from Sham group maintained the memory of the task and this was significantly higher than the memory of the Nx group [F_(1,129)_ = 5.2, P < 0.05]. Nx group showed worse performance during all observational time (B). *: P < 0.05; *a*: P < 0.001. The group was divided into Nx-M group (latencies above the median level) and Nx-Cl group (latencies under the median level) (C).

In the first exposure of animals to the inhibitory avoidance equipment, Sham and Nx groups entered the dark space in short time, showing no difference concerning the preference for this ambient [20.8 sec (11.8/26.1) sec, n = 12 and 13.2 sec (7.3/18.5) sec, respectively]. However, a significant difference between both groups behavior was observed in the test performed 24h after the training session (Fig 3A). Sham group showed a significant increase in latency to enter the dark room [103.9 sec (36.1/168.2) sec, (F_1,51_ = 23.1, P < 0.001], suggesting that they remembered the task. However, Nx group presented a latency time similar to the time in the training session [28.6 sec (16.6/62.0) sec], suggesting that they did not retained the information. The performance of Nx group in the 24h-test session was 27.5% lower than Sham group (F_1,51_ = 5.4, P < 0.05) (Fig 3A).

During the observational time, 24h-test was considered the 0 (zero) time. Animals from Sham group maintained the memory of the task and this was significantly higher than the memory of the Nx group [F_(1,129)_ = 5.2, P < 0.05]. Besides, retesting improved memory of both groups, as Sham and Nx animals showed increased latencies along the 120 days [F_(1,129)_ = 6.7, P < 0.001], but Sham group had better memory in 30 (P < 0.01) and 60 days (P < 0.05). Nx performance at the end of the protocol was not homogenous [93.8 sec (18.2/180.0], different from the observed in Sham group [180.0 sec (102.3/180.0) sec] (Fig 3B). So, Nx animals that presented latencies above the median level were included in the Nx-memory (Nx-M) group (n = 7), while the other Nx rats with scores under the median level formed the Nx-CI group (n = 7).

**Fig 4:**
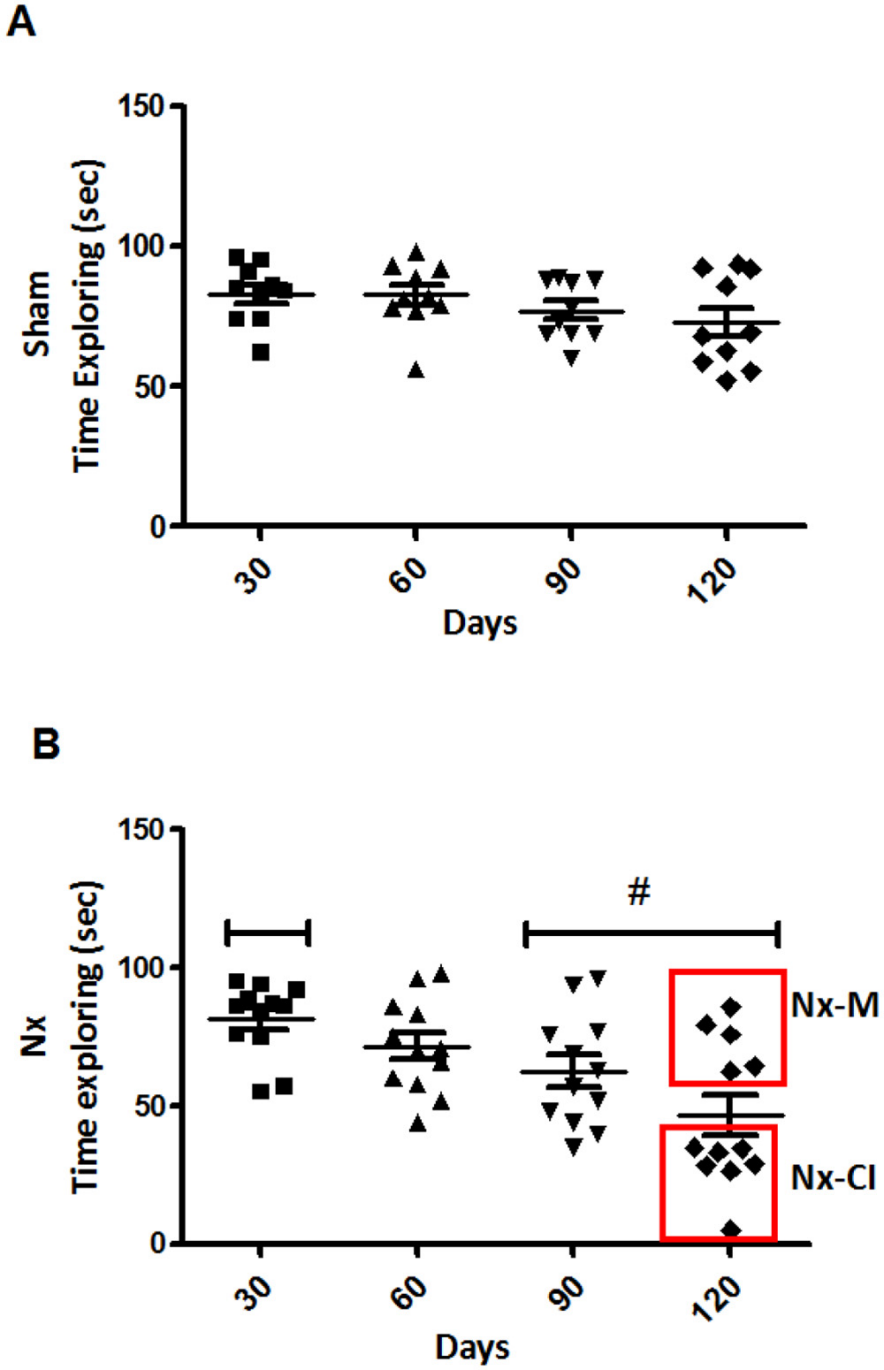
Novel Object Recognition Test analysis in sham and Nx. All the animals were tested at 30, 60, 90 and 120 days following the training day. Kruskal-Wallis one-way ANOVA (22.67, p<0.001) with Müller-Dunn post-tests: Nx-90, Nx-120 vs Nx-30, p<0.005. Nx group was divided into two groups: those animals that remember the task (Nx-M group, n = 5) and those animals that did not remember the task (Nx-Cl, n = 7) (red boxes). Student's t test, p <0.05. Both tests showed that Nx induced memory impairment.

By using the novel object recognition test, a non-aversive environment, we found that all sham rats explored the new object for at least 73% of total time (Fig 4A) during all the observational time (120 days). However, the mean spending time exploring the new object for the Nx group decreased along time and, at 120 days, these groups spent 46.7% of total time doing the exploration (Fig 4B). Regarding these observations, Nx group was divided into two groups: those animals that remember the task (Nx-M group, n = 5) and those animals that did not remember the task (Nx-Cl, n = 7). It is suggested that even in the absence of an aversive stimulus, 58% of Nx rats had an attention deficit and were unable to identify the new object (Fig 4B).

**Fig 5:**
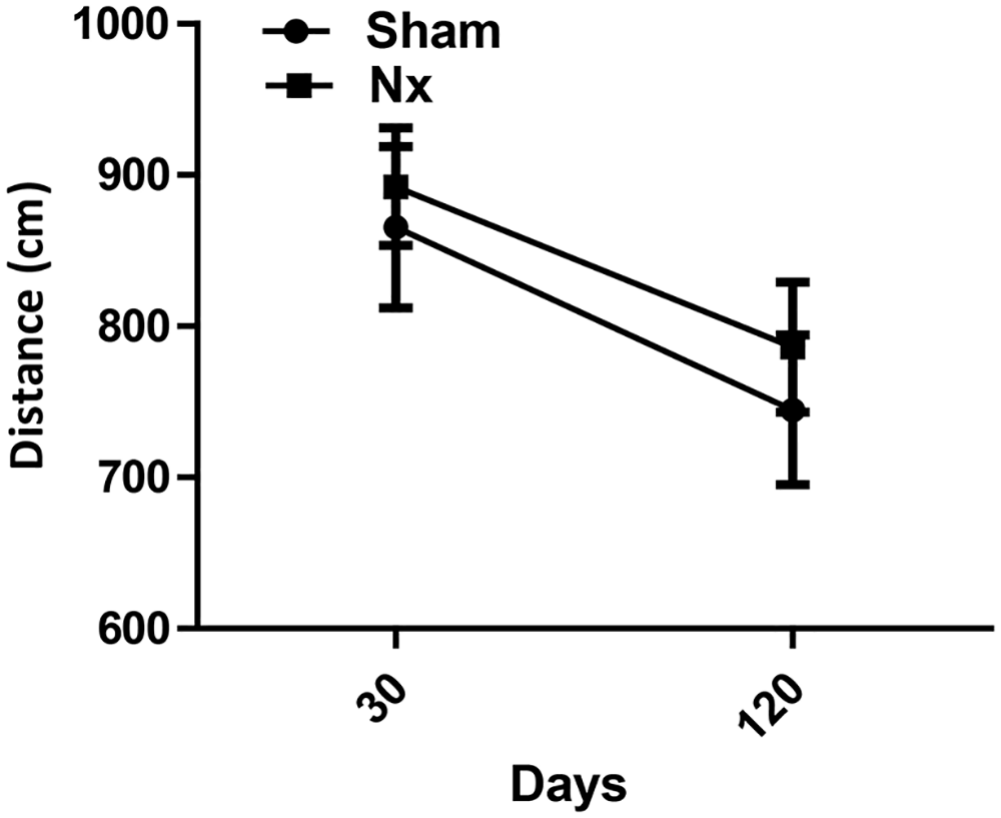
Locomotor activity in sham and Nx. Total distance travelled during 300s of Sham (n = 20) and Nx (n = 28) groups at 30 and 120 days—Kruskal-Wallis one-way ANOVA with Müller-Dunn post-test, F(1,3) = 0.17, NS.

Results showed no significant difference between the locomotor performance in sham (horizontal; vertical) and Nx (horizontal; vertical) groups after five minutes testing in the apparatus at 30 and 120 days after surgery, suggesting that the differences found in cognition are not due to decreased movement within the environment (Fig 5).

### Nx-groups induced NF-κB activation in rat hippocampus and frontal cortex

**Fig 6:**
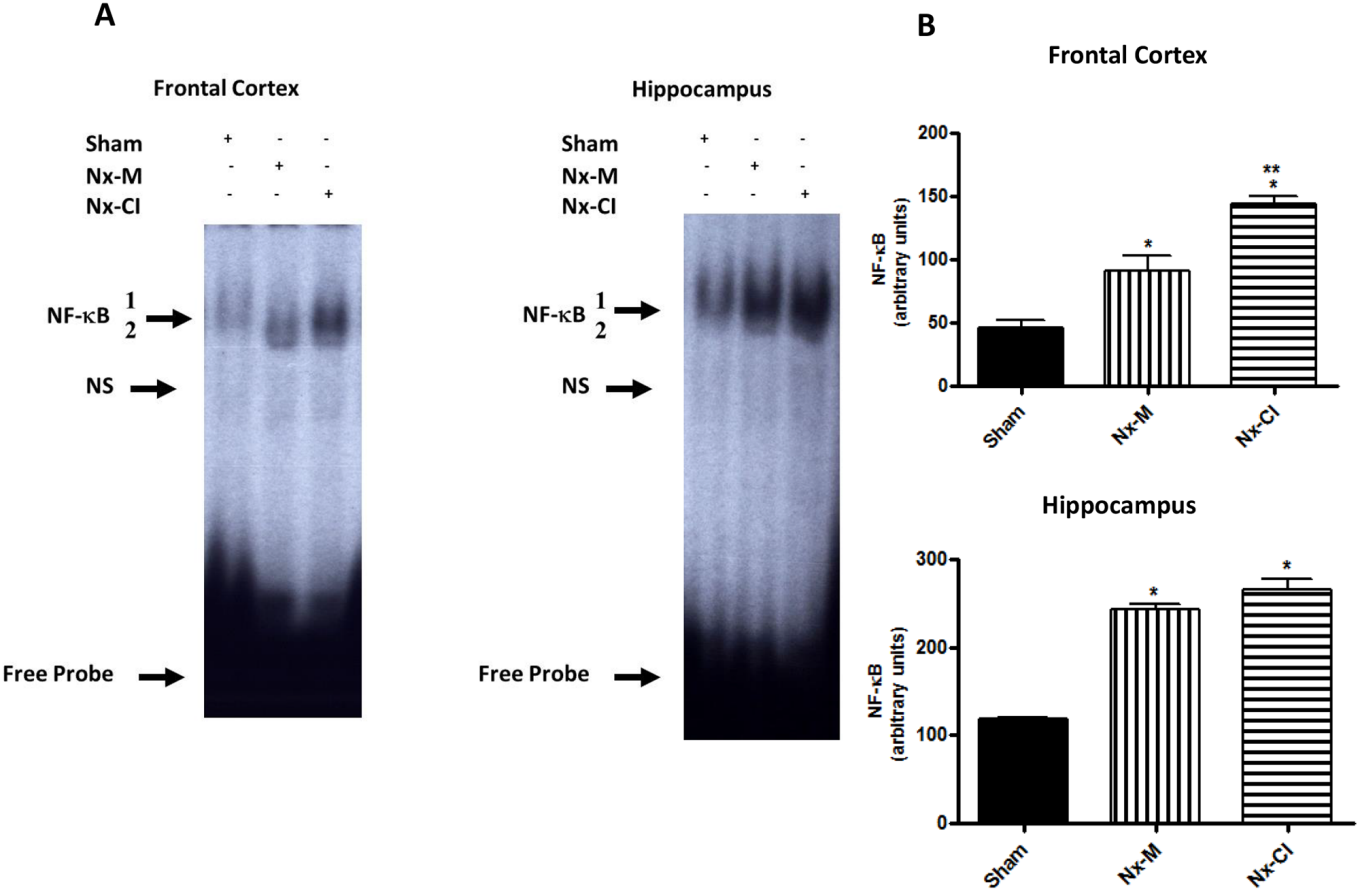
Changes in NF-κB activity in rat hippocampus and frontex cortex at 121 days in sham and Nx (Nx-M and Nx-CI) groups. (*A*) Nuclear proteins (15 μg) were extracted from frontal cortex and hippocampus of sham, Nx-M and Nx-CI groups. (*B*) Densitometric analysis (arbitrary units, A.U.) of the NF-κB bands (complexes 1 and 2) are presented in panel A. Results are expressed as mean ± S.E.M. from three individual experiments (n = 9). *p<0.05 versus sham group and **versus Nx-M group—one-way ANOVA followed by Newman-Keuls test. Frontal Cortex: F (2,6) = 31.76, p<0.001; Hippocampus F(2,6) = 106.7, p<0.0001. The positions of specific NF-κB/DNA and non-specific (NS) binding complexes are indicated.

EMSAs were performed to study the effects of Nx on NF-κB binding activity in the rat hippocampus and frontal cortex. EMSA analyses showed that both Nx-groups had increased NF-κB binding activity but the activation of this transcription factor was higher in frontal cortex of the Nx-CI animals (Fig 6A and 6B). Nuclear extracts from the Nx-groups tissues presented a similar pattern of DNA/protein complexes from our previous control samples. The upper complexes 1 and 2 were displaced by an excess of unlabeled NF-κB, but not TFIID double-stranded oligonucleotide consensus sequence, demonstrating the specificity of the NF-κB/DNA binding interaction. The lower complexes were less efficiently displaced by the unlabeled NF-κB probe. Supershift analysis confirmed previous data from our laboratory [29] indicating that the antibody against the subunit RelA was able to shift DNA/protein interaction present in complex 1. The antibody against the subunit p50 shifted complex 2 and induced a partial decrease in complex 1. The presence of antibodies against the subunits cREL and p52 (data not shown) did not affect DNA—protein complexes. Taken together, these results indicate that p50/RELA heterodimers and p50/p50 homodimers were included in ^32^P-NF-κB/protein complexes 1 and 2, respectively. Complex 3 was not displaced by the antibodies and was not considered related to NF-κB family signaling processes [28].

### Nx-groups induced Oxidative Stress in rat cerebellum

Both Nx groups (nx-M and NX-CI) have higher TBARS levels and neuronal (nNOS) and inducible (iNOS) activities in rat cerebellum when compared to the control group. However, the levels of TBARS and NOS enzyme activity were higher in Nx-CI animals when compared to Nx-M group suggesting that other brain areas are also exposed to oxidative stress and neuroinflammation (S1 Fig and S2 Fig).

### Tissue and CSF levels of cytokines in Nx-M and Nx-CI groups

**Fig 7:**
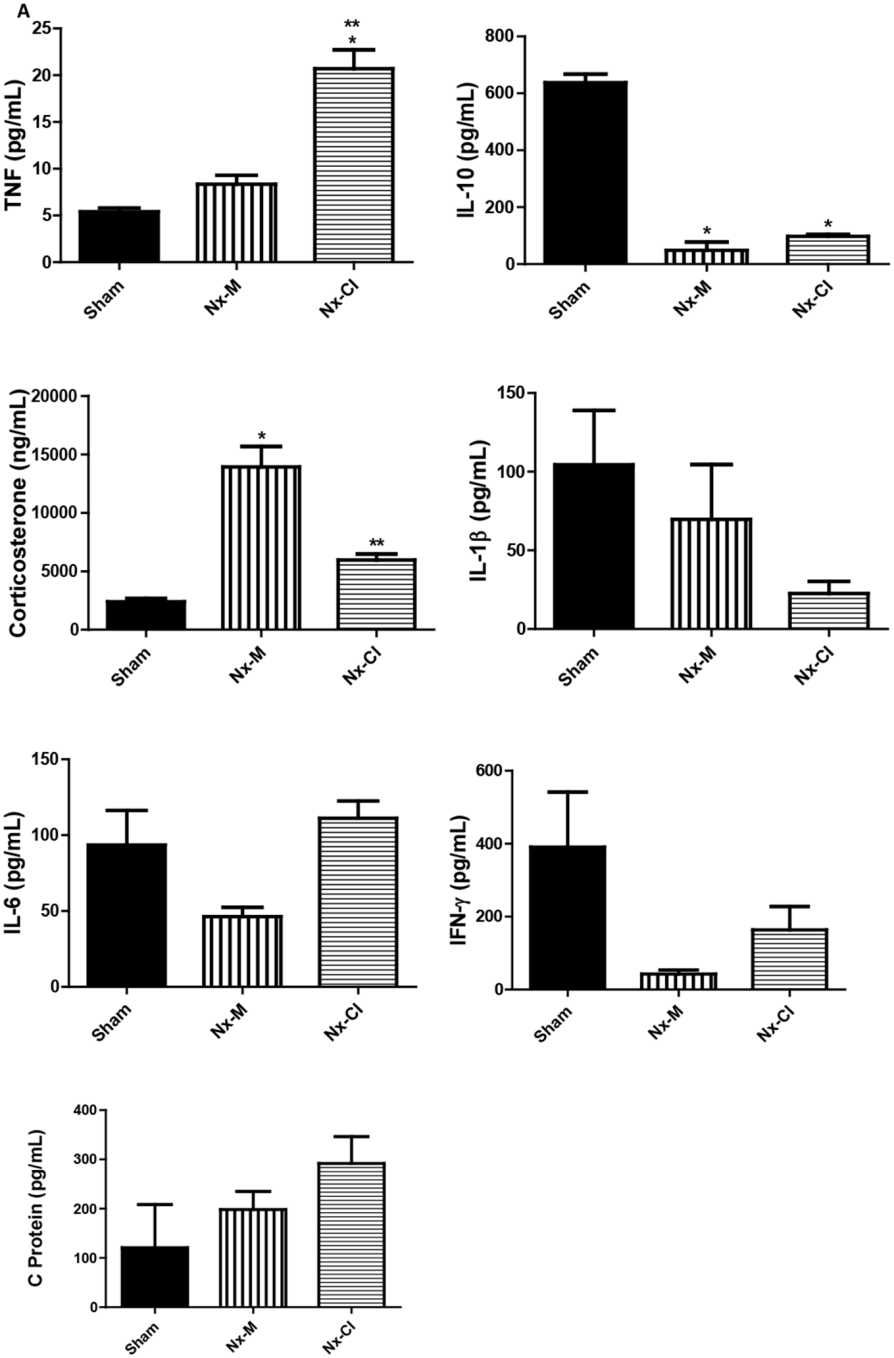
Changes in TNF-α, IL-10, Il-1β, IL-6, IFN-γ, CRP and GC in CSF at 121 days in sham and Nx (Nx-M and Nx-CI) groups. Values are the mean ± S.E.M. (n = 21). *p<0.05 versus sham group and ** versus Nx-M group—one-way ANOVA followed by Newman-Keuls test. TNF-α F(2,6) = 38.75, p<0.001; IL-10 F(2,7) = 200.50, p<0.0001; GC F(2,6) = 30.75, p<0.001; IL1-β F(2,6) = 38.75, p<0.001; IL1-β F(2,11) = 1.62, p<0.001; IL6 F(2,9) = 2.78, NS; IFNγ F(2,8) = 2.75, NS; CRP F(2,3) = 1.83, NS.

**Fig 8:**
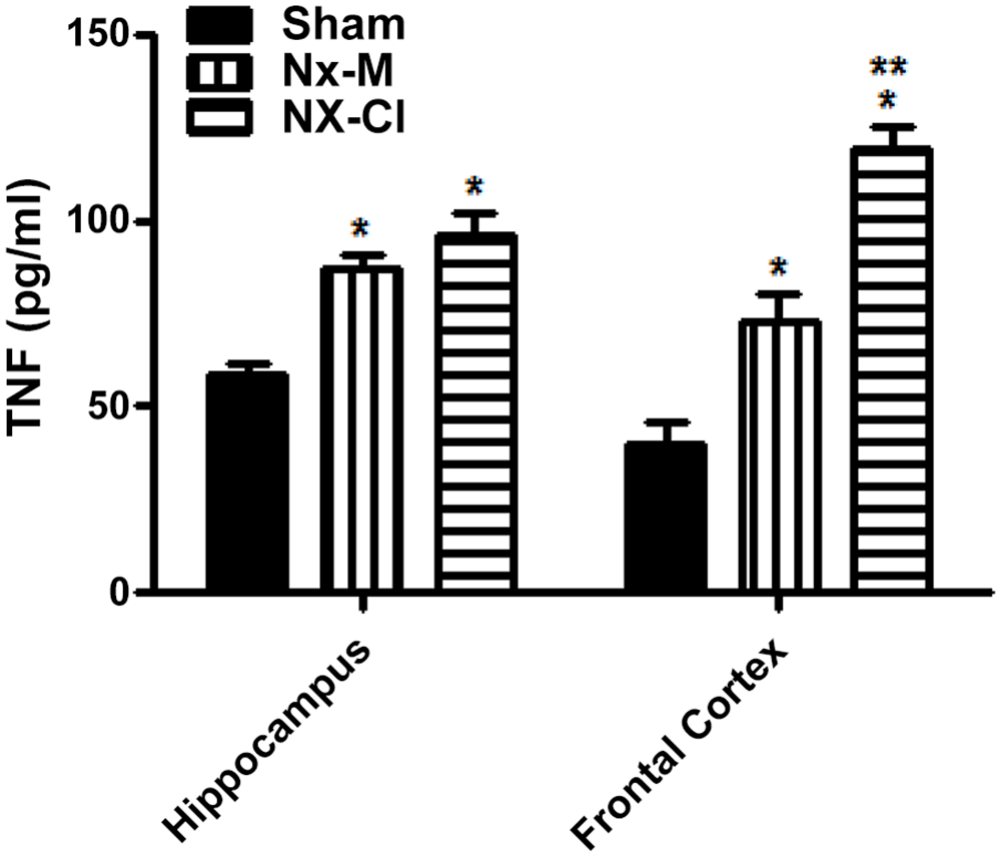
Changes in TNF-α in hippocampus and frontal cortex at 121 days in sham and Nx (Nx-M and Nx-CI) groups. Values are the mean ± S.E.M. (n = 21). *p<0.05 versus sham group and ** versus Nx-M group—one-way ANOVA followed by Newman-Keuls test. TNF-α Frontal cortex: F(2,14) = 39.43, p<0.001; TNF-α hippocampus: F(2,14) = 9.24, p<0.05.

**Fig 9:**
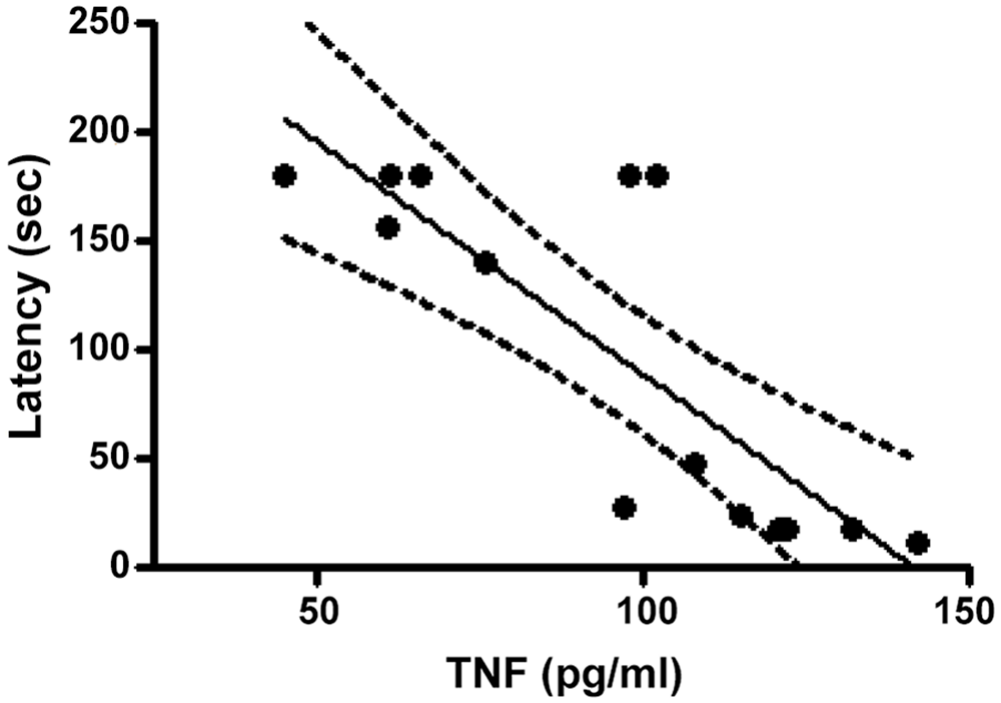
Correlation linear and regression between values of latency time measured in the Inhibitory avoidance test analysis at 120 days of observation and TNF-α in frontal cortex at 121 days in Nx (Nx-M and Nx-CI) groups. Analyses were performed based on [70] by using GraphPad Prism Software. Pearson correlation coefficient *r* = −0.67, *p* < 0.001; linear regression, *r*2 = 0.67, *F* = 24.7, *p* < 0.001.

The CSF data showed that the Nx-CI group had a clear pro-inflammatory status, as indicated by high TNFα levels and low IL-10 levels versus the Nx-M and Sham groups. Although there were no significant differences in CSF TNFα levels between the Nx-M and Sham groups, a trend increase in TNFα levels in the Nx-M group suggests that the level of TNF-α increase may be variable following Nx and linked to CI levels. Concurrently, the anti-inflammatory cytokine, IL-10, was lower in the CSF in both Nx (Nx-M and Nx-CI) groups than in the Sham group. IL-1β levels were lower in the Nx-CI group compared to Sham and Nx-M groups, whilst the Nx-M group had lower lL-6 and IFNγ levels when compared to sham and Nx-CI groups (Fig 7). Furthermore, both Nx groups (Nx-M and NX-CI) showed increased CSF GC levels when compared to sham group, with the Nx-M group exhibiting higher levels than the Nx-CI group. Serum and CSF CRP levels showed no significant difference across all groups studied (sham, Nx-M and Nx-CI) (Fig 7). As this data indicate a possibility of inflammatory pattern linked to TNFα in the CSF of Nx-CI rats, we evaluated changes induced by Nx on hippocampus and frontal cortex TNFα levels. Although levels of TNFα were increased in the hippocampus and frontal cortex of both Nx-groups when compared to the Sham group, Nx-CI animals showed higher frontal cortex levels of this cytokine (Fig 8). In addition, there is a trend negative correlation between increased frontal cortex TNFα and decreased latency scores, indicating the role of frontal cortex TNFα in the CI evident in the inhibitory avoidance test. In addition, TNFα and latency scores negatively covary (Fig 9).

### Nx-CI decreases frontal cortex KLOTHO levels

**Fig 10:**
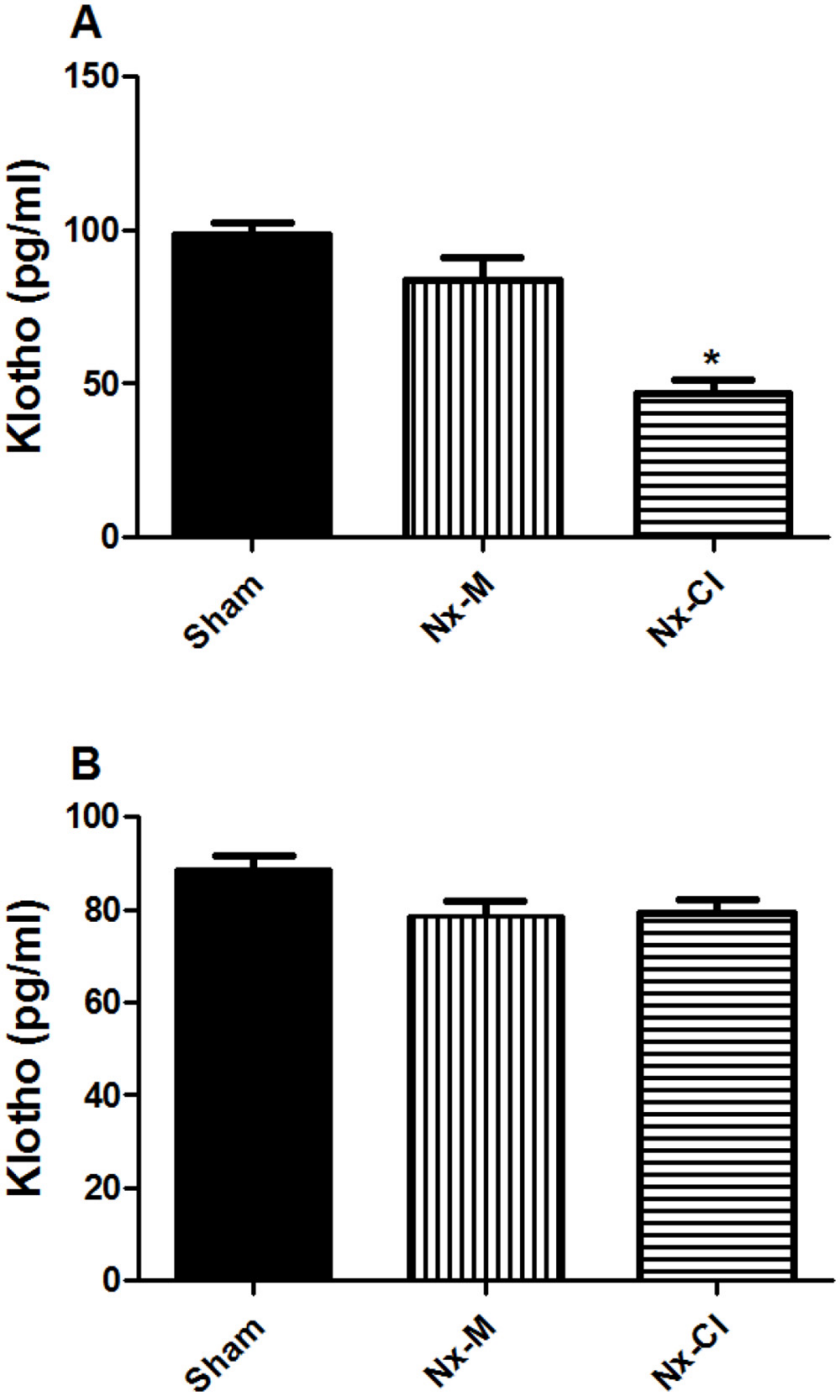
Changes in soluble KLOTHO levels in rat (A) frontal cortex and (B) hippocampus at 121 days in sham and Nx (Nx-M and Nx-CI) groups. Values are the mean ± S.E.M. (n = 21)—*p< 0.05 vs control and Nx-M—one way ANOVA followed by Newman-Keuls test. Frontal cortex: F(2,18): 23.23, p<0.0001; Hippocampus: F(2,12): 3.79, NS.

**Fig 11:**
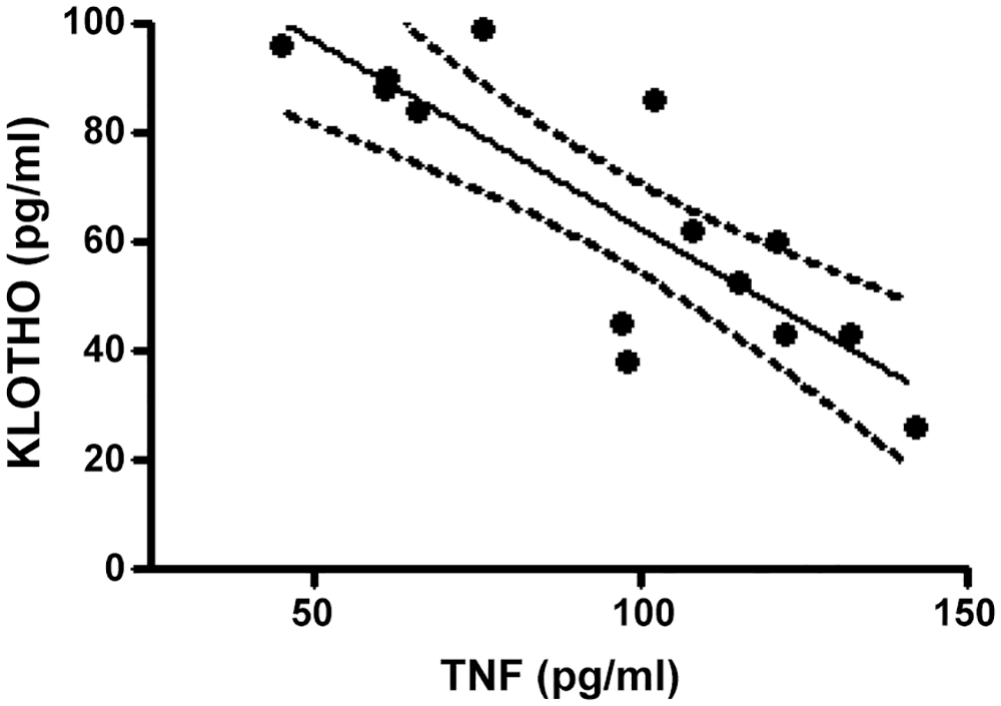
Correlation linear and regression between values of TNF-α and KLOTHO levels in frontal cortex at 121 days in Nx (Nx-M and Nx-CI) groups. Analyses were performed based on [70] by using GraphPad Prism Software. Pearson correlation coefficient *r* = −0.70, *p* < 0.001; linear regression, *r*2 = 0.70, *F* = 28.3, *p* < 0.001.

The soluble KLOTHO assay showed a tendency for decreased protein levels in all Nx-groups, with a significant decrease only evident in the Nx-CI group frontal cortex (Fig 10). In addition, the correlation between basal TNFα and KLOTHO levels in Nx-CI animals showed a negative association with frontal cortex TNFα and KLOTHO levels. Results also showed that TNFα and KLOTHO variables covary (Fig 11).

## Discussion

Recent research has shown that the degree of CI is associated with the level of renal functional impairment. It is believed that these cognitive changes are related to vascular changes in the CNS due to CKD, as well as to changes triggered by hemodialysis procedures [30, 31]. It is well known that the immune system plays a central role in modulating learning, memory and neural plasticity [32]. In conditions where the immune system is strongly activated, as in the end stage of renal diseases [33], the production of these inflammatory mediators disrupts the delicate balance needed for the normal neurophysiological actions of immune processes and produces direct detrimental effects on memory, neural plasticity and neurogenesis [32].

In the present work we evaluated the correlations of CI with inflammatory response and soluble KLOTHO in hippocampal and frontal cortex samples of experimental model of 5/6 nephrectomy (Nx) rats. Nx is one of the most used animal models of progressive renal failure by reduced nephron number, best-characterized in rats. The reduction of renal mass is achieved by either infarction or surgical excision of both poles, with removal of the contralateral kidney. The initially normal remnant nephrons undergo compensatory functional and structural adaptations. Simultaneous glomerular hypertension is one of the main factors responsible for the development of renal injury. Morphologicaly progressive focal segmental to global glomerulosclerosis is present, accompanied clinically by increasing proteinuria and deteriorating renal function [34]. In fact, all Nx animals exhibited an increase in the percentage of sclerotic glomeruli and an expansion of the cortical interstitial area at 121 days after the surgery confirming the progressive renal failure (Fig 2).

Firstly, we evaluated the behavioral changes induced by Nx, indicative of CI. Data revealed a decline in working memory and attention in an Nx animal model, suggesting that CKD, even in its earliest stages, contributes to the occurrence of CI. This may be of clinical relevance, given that the equivalent stage in human CKD is not thought to warrant the use of renal replacement therapy.

The step-through inhibitory avoidance test is based on the natural dark-seeking behavior of rats and evaluates long-term, declarative memory. It is a fear-motivated task in which the rat refrains from stepping through a door to an apparently safer dark compartment previously linked to punishment [35, 36]. Our results showed that 50% of animals from the Nx group (Nx-CI) did not remember the aversive stimulus (foot shock) at 90 days after Nx surgery and at the last re-test (120 days) after of the training phase in an inhibitory avoidance test when compared to both Nx-M and sham groups. The presence of memory impairment was confirmed by using the novel object recognition test, which is a relatively simple and straightforward method to test rodent working memory [25]. Results showed that 61% of the animals from the Nx group exhibited a deficit of attention and new object recognition. Therefore, our data show for the first time that the Nx animal model induces working memory and attentional impairments, which were not due to changes in the ability of these animals to explore the environment, since no differences were found in locomotor activity versus both Nx-M and sham groups.

Neuroinflammatory conditions such as traumatic brain injury, Alzheimer’s disease, Down’s syndrome and aging are frequently associated with CI [37–39], and there is considerable evidence for an association between cytokine expression in the brain and CI, including memory deficits [40–42]. For instance, it has been shown that TNFα and IL-6 are critical for neuroinflammation induced memory impairment [43–45]. Data obtained here, in the study of animals undergoing ablation of 5/6 renal mass, showed that changes in CSF levels of inflammatory mediators may be linked to CI-induced by CKD.

NF-κB plays roles in memory and neuroinflammation, suggesting that its nuclear transcription factor function is part of a complicated response that is involved in long term memory and cellular plasticity [46, 47]. Analysis of NF-κB by EMSA showed that NF-κB was increased in both Nx-groups in frontal cortex and hippocampus, with its activation being higher in frontal cortex of Nx-CI animals versus Nx-M and sham groups.

In addition, although Nx-CI and NX-CM groups exhibited an increase in TBARS levels and NOS (nNOS and iNOS) activities when compared to control group in rat cerebellum, the levels of TBARS and NOS enzyme activity were more elevated in Nx-CI when compared to Nx-CM animals. These data suggest the presence of more intense oxidative stress in Nx-CI animals which could be relevant to CI induced by chronic inflammation status.

A correlation between inflammation and the presence of CI is further indicated by the specific increase in CSF TNFα levels in the Nx-CI group compared to Nx-M, suggesting a significant role for this cytokine in the CI induced by CKD. In support of this, Nx—induced an increase of TNFα levels in both frontal cortex and hippocampus, with the levels of this cytokine being higher in the frontal cortex of the Nx-CI group when compared to the Nx-M group. The Pearson’s analysis showed a significant negative correlation between increased frontal cortex TNFα and decreased latency scores in the inhibitory avoidance test, highlighting a correlation between neuroinflammation and CI. In addition, Nx groups (Nx-M and Nx-CI), when compared to the sham group, showed lower levels of IL-10, a cytokine that is known to counteract NF-κB pro-inflammatory signaling [48]. The pro-inflammatory cytokine TNFα plays a central role in the onset and maintenance of inflammation [49], while IL-10 is a prototypical anti-inflammatory cytokine that inhibits the production of inflammatory cytokines and chemokines, including IL-1β, IL-6, TNFα, IFNγ and RANTES [50, 51]. Therefore, an increase in the TNFα/IL-10 ratio could be linked to CI by the maintenance of NF-kB activation in the Nx groups.

TNFα is one of the key mediators of renal injury, driven by NF-KB induced transcriptions [5]. TNFα plays complex roles in the CNS, depending upon which TNF receptor is engaged [52]. Whilst activation of TNFR2 is neuroprotective, activation of TNFR1 signaling pathways is generally linked to apoptosis, including via increased microglia glutamate release, which drives excitotoxicity and CI [53].

Generally, GCs are associated with anti-inflammatory effects, which could explain the maintained cognition in the Nx-M group, where increased CSF GCs were evident. However, there are circumstances following an inflammatory challenge or insult where GCs or stress enhance rather than blunt inflammation [54]. Consonant with this, we have shown that chronic unpredictable stress (CUS) potentiates lipopolysaccharide (LPS)-induced NF-κB activation and proinflammatory cytokine expression in the frontal cortex and hippocampus, but not in hypothalamus of rats. The proinflammatory effects of stress were mediated by GC receptors since pretreatment with the GR antagonist RU-486 blunted the stress effects on NF-κB potentiation [29]. As such, GCs may have variable effects on inflammatory processes, dampening inflammation in some circumstances, whilst enhancing inflammation in other circumstances, at least in part via the sensitization of microglia reactivity, overlapping with the detrimental effects of stress [55].

Interestingly, data show a relationship between increased inflammatory processes with increased levels of depression in CKD [56]. Also recent data suggest that the cardiovascular disease (CVD) associated with CKD may be driven, at least partly, by products derived from the tryptophan pathway, such as kynurenic and quinolinic acid [57]. Increased pro-inflammatory cytokines, such as TNF-α and IL-6, can activate IDO (indoleamine 2,3-dioxygenase), with impacts on mood, CVD and cognition [58]. Decreased IFNγ in the Nx-M group could also be linked to decreased IDO, given that IFNγ is the major IDO inducer. In this context, increased levels of central GC, which can induce TDO (tryptophan 2,3-dioxygenase), may also increase levels of tryptophan catabolites. As such, the tryptophan catabolite pathways may integrate and partly mediate the changes driven by increased pro-inflammatory cytokines and GC in the Nx-CI and Nx-M respectively.

Short-term GCs, as with immune activation, can be beneficial, whereas long-term GCs and prolonged or exaggerated inflammation can have adverse consequences [59]. It is of note in this context that KLOTHO has an anti- inflammatory action and a renoprotective effect in renal diseases through the inhibition of NF-kB activation. This subsequently inhibits inflammatory cytokine production, including in protective response to TNF-α stimulation in kidney cells, which is mediated by inhibiting p38 kinase and specifically blocking the phosphorylation of Ser536 of p65, without affecting I-κB, IκBα degradation or the translocation of p65 to the nucleus and its DNA binding [60, 61]. The interactions among KLOTHO, TNFα, NF-kB and inflammatory processes are not restricted to the kidney, with studies suggest that endothelial dysfunction observed in mice deficient in KLOTHO is reversed by KLOTHO reposition [7, 62]. Endothelial function is mediated by the regulation of nitric oxide and is impaired by inflammatory processes [24]. Thus, KLOTHO appears to protect endothelial cells from the inflammatory process, suppressing the development of atherosclerosis in the vascular system.

Cognitive function measured by novel-object recognition and conditioned-fear tests in *KLOTHO* mutant mice was normal at the age of 6 wk, but markedly impaired at the age of 7 wk [63]. Results also showed that at the age of 7wk the KLOTHO mutant mice exhibited in the hippocampus increased levels of oxidative stress and pro-death Bax whereas anti-death Bcl-2 and Bcl-XL levels are decreased, and apoptotic TUNEL-positive cells were detected [63]. In addition, Li et al. [18] demonstrated that synaptic structures and synaptophysin are reduced in number and expression, respectively, in the CA3 region of KLOTHO mutant mice. Recent data also suggest that inactivation of the JAK2/STAT3 signaling axis and M1 mAChR downregulation play a critical role in cognitive impairment observed in KLOTHO mutant mice [64].

In our study we found a decrease in the protein levels in the frontal cortex of Nx-CI animals compared with Nx-M and Sham. Relevant to this observation is the significantly increased TNF-α levels and NF-kB activation in the same brain area, when compared to Nx-M and sham groups. Studies from our laboratory showed that Hemodialysis (HD) patients without cognitive deficit show an increase in *TNF-α serum levels*. In addition, there is a correlation of high *TNF-α* with a presence of low serum Klotho levels in HD patients with cognitive deficit as evaluated by Modified Mini Exam of Mental State (3MS) and Kidney Disease Quality of Life (KDQOL-sf) (unpublished data). This suggests an interaction between KLOTHO and inflammatory signaling over the course of CI, which is supported by the Pearson’s correlation showing a significant negative association of TNFα and KLOTHO levels in frontal cortex of Nx groups.

In fact, studies have shown that TNFα inhibits the expression of β-KLOTHO, in turn preventing the action of FGF-21 in adipocytes [65]. Recent evidence showed that recombinant Klotho protected rat primary hippocampal neurons and hippocampal neuronal cell line TH22 from glutamate and oligomeric amyloid β-induced cytotoxicity by regulation of members of the redox system [66]. In addition, data show that a lifespan-extending variant of the human KLOTHO gene, KL-VS, is associated with enhanced cognition in heterozygous carriers and transgenic mice with systemic overexpression of KLOTHO performed better than controls in multiple tests of learning and memory [67]. Raising KLOTHO levels in mice also increases synaptic plasticity associated long-term potentiation, driven by increased synaptic GluN2B, an N-methyl-D-aspartate receptor (NMDAR) subunit with key functions in learning and memory [67]. As KLOTHO can increase sirtuin-1, which has both memory and longevity benefits via mitochondrial regulation, as well as inhibiting TNFα effects, some of the efficacy of KLOTHO overexpression may be driven by sirtuin upregulation [68, 69]. The role of sirtuins in the effects of KLOTHO, including in decreasing TNFα, requires further investigation in the context of cognitive function at different life stages. Overall, understanding the TNFα–KLOTHO interaction and their interactions with other inflammatory mediators, neurotransmitters and hormones, as well as metabolic and growth factors in the CNS will be important in the development of new strategies to delay the onset of memory loss and CI linked to CKD and perhaps neurodegenerative diseases more widely.

## Supporting Information

S1 Fig Changes in TBARS in rat cerebellum at 121 days in sham and Nx (Nx-M and Nx-CI) groups. Lipid peroxidation was determined through the production of TBARS, as previously described [71]. Hippocampi tissues were homogenized in saline buffer and precipitated proteins were removed by centrifugation at 12,000xg for 10 min. The supernatant was mixed with thiobarbituric acid (1% in NaOH 50 mm) and HCl 25%. The samples were then heated in a boiling water bath for 10 min and, after cooling, were extracted with 1.5 mL of butanol. The mixture was centrifuged at 12,000xg for 10 min and the absorbance of the supernatant was determined [72]. Values are the mean ± SEM. Statistical analysis: One-way ANOVA followed by Newman—Keuls test: F (2,21) = 61.2, p< 0.0001; a vs Sham, b vs NX-M, p<0.001.
(TIF)

S2 Fig Changes in (A) nNOS and (B) iNOS activities at 121 days in sham and Nx (Nx-M and Nx-CI) groups. For NOS activity assay, the tissue samples were homogenized in ice-cold 0.32 M sucrose/20 mM HEPES buffer (pH 7.4) containing 1 mM dithiothreitol (DTT) in an ice bath for 1 min using a Teflon homogenizer. Each homogenate was centrifuged at 10,000×g for 30 min at 4°C. The supernatant was passed through a Dowex AG 50 Wx-8 (Na^+^ form) column to remove the endogenous arginine. The arginine-free eluent was used to assay the NOS activity. NOS activity in cerebellum was determined by the enzymatic conversion of [^3^H]arginine to [^3^H]citrulline as described by [73] with some modifications. Briefly, the NOS assay reaction medium of 200 μL, containing 100 mM HEPES, pH 7.4; 1 mM NADPH; FMN, FAD, Tetrahydrobiopterin 0.45 mM CaCl_2_; 80 units of calmodulin, 100 μM L-arginine, and 1 μM L-[2,3-^3^H]-arginine (0.5 μCi), or with no addition of CaCl_2_ and calmodulin (in the presence of 0.425 mM EDTA), and 100 μL of hippocampus cytosolic protein (0.2 μg/μL). The reaction mixture was incubated for 30 min at 37°C and stopped by the addition of stop buffer containing 20 mM HEPES at pH 5.5. The entire reaction mixture was passed through a column packed with Na^+^ form of Dowex AG 50 Wx-8 resin. The flow through fraction containing [^3^H]-citrulline was counted for radioactivity using a Beckman 6000 liquid scintillation counter. The NOS activity was expressed as picomoles citrulline per milligram protein per minute. Inhibition of the enzyme was evaluated in all tissues using N-nitro-L-arginine methyl ester hydrochloride (L-NAME—10^-6^ to 10^-4^ M). The biochemical characterization of cerebellum NOS showed that spontaneous activity of NOS in the cytosol of hippocampus tissue was greatly reduced when NADPH or Ca^2+^/ calmodulin was omitted from the incubations medium for the convertion of L-arginine to L-citrulline. The biochemical characterization data of the constitutive NOS isoform in the cerebellum confirmed previous data from our laboratory as Ca^2+^-independent form of NOS (iNOS activity) represents < 3% of total NOS activity in the cerebellum [74]. Values are the mean ± SEM. Statistical analysis: One-way ANOVA followed by Newman—Keuls test: (A): F (2,20) = 71,7, p< 0.0001; a vs Sham, b vs NX-M, p<0.001. (B): F (2,20) = 58,7, p< 0.0001; a vs Sham, b vs NX-M, p<0.0001.
(TIF)
