# Supplementary figures and images for: Altered KLOTHO and NF-κB-TNF-α Signaling Are Correlated with Nephrectomy-Induced Cognitive Impairment in Rats
Source: PLoS One. 2015 May 11;10(5):e0125271. doi: 10.1371/journal.pone.0125271 (PMC4427267; doi:10.1371/journal.pone.0125271)

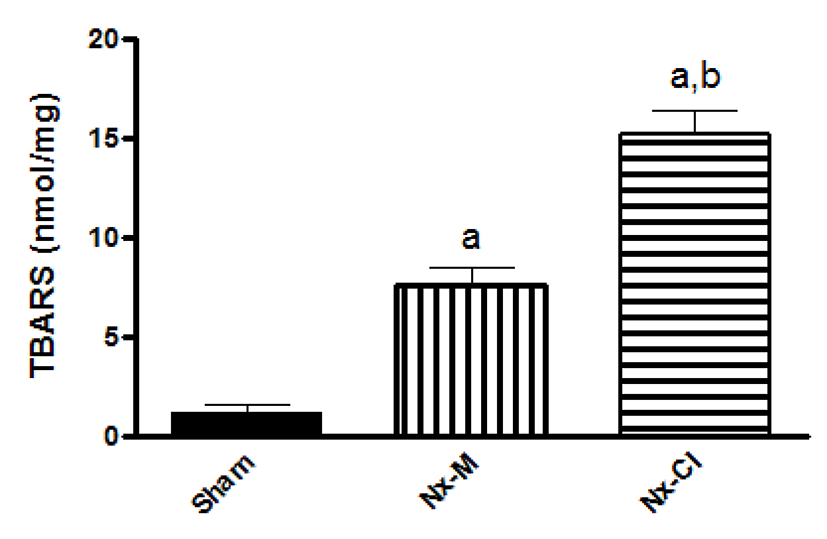

Supplement: S1 Fig — Lipid peroxidation was determined through the production of TBARS, as previously described [71]. Hippocampi tissues were homogenized in saline buffer and precipitated proteins were removed by centrifugation at 12,000xg for 10 min. The supernatant was mixed with thiobarbituric acid (1% in NaOH 50 mm) and HCl 25%. The samples were then heated in a boiling water bath for 10 min and, after cooling, were extracted with 1.5 mL of butanol. The mixture was centrifuged at 12,000xg for 10 min and the absorbance of the supernatant was determined [72]. Values are the mean ± SEM. Statistical analysis: One-way ANOVA followed by Newman—Keuls test: F (2,21) = 61.2, p< 0.0001; a vs Sham, b vs NX-M, p<0.001. (TIF) [file pone.0125271.s001.tif]

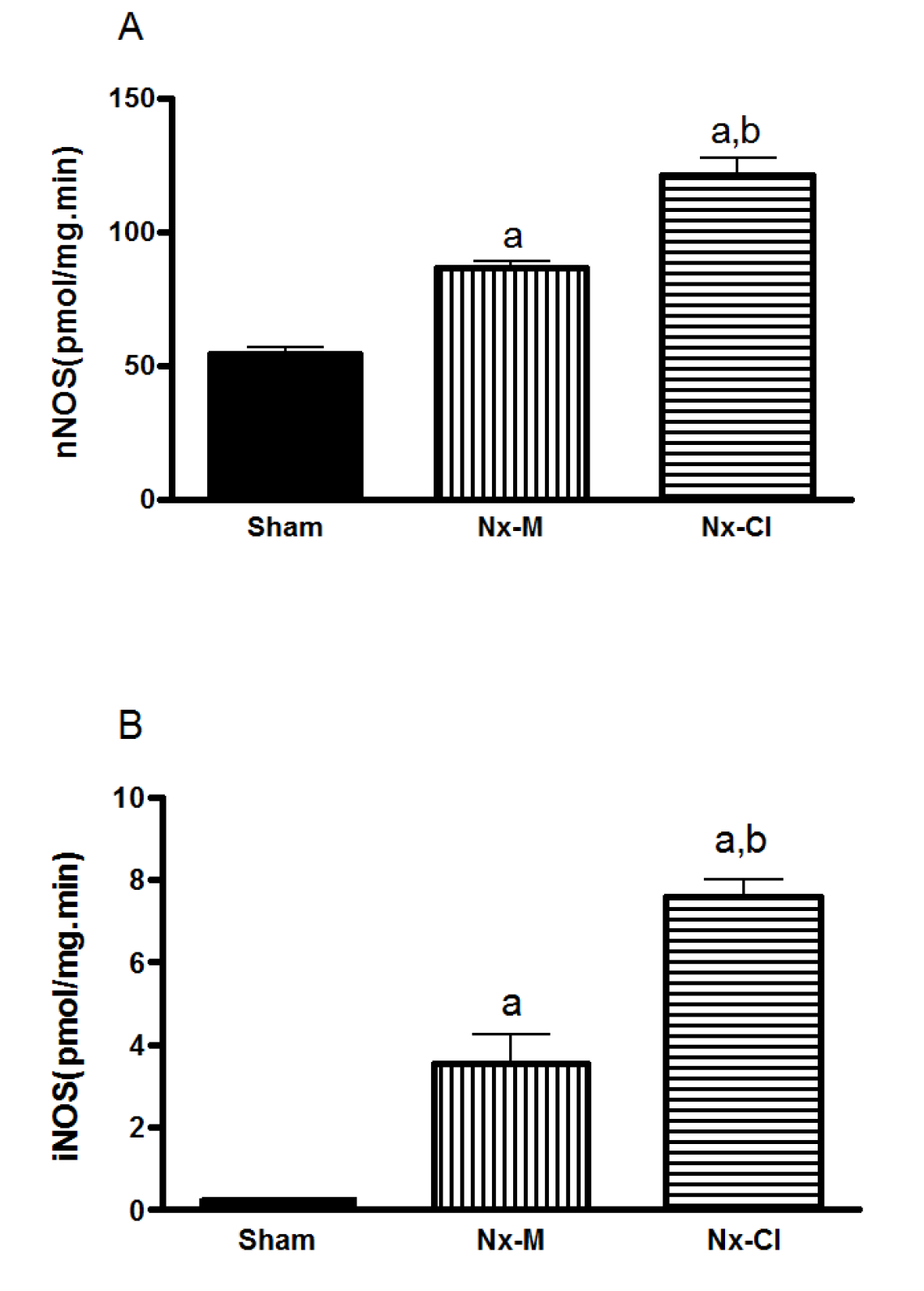

Supplement: S2 Fig — For NOS activity assay, the tissue samples were homogenized in ice-cold 0.32 M sucrose/20 mM HEPES buffer (pH 7.4) containing 1 mM dithiothreitol (DTT) in an ice bath for 1 min using a Teflon homogenizer. Each homogenate was centrifuged at 10,000×g for 30 min at 4°C. The supernatant was passed through a Dowex AG 50 Wx-8 (Na+ form) column to remove the endogenous arginine. The arginine-free eluent was used to assay the NOS activity. NOS activity in cerebellum was determined by the enzymatic conversion of [3H]arginine to [3H]citrulline as described by [73] with some modifications. Briefly, the NOS assay reaction medium of 200 μL, containing 100 mM HEPES, pH 7.4; 1 mM NADPH; FMN, FAD, Tetrahydrobiopterin 0.45 mM CaCl2; 80 units of calmodulin, 100 μM L-arginine, and 1 μM L-[2,3-3H]-arginine (0.5 μCi), or with no addition of CaCl2 and calmodulin (in the presence of 0.425 mM EDTA), and 100 μL of hippocampus cytosolic protein (0.2 μg/μL). The reaction mixture was incubated for 30 min at 37°C and stopped by the addition of stop buffer containing 20 mM HEPES at pH 5.5. The entire reaction mixture was passed through a column packed with Na+ form of Dowex AG 50 Wx-8 resin. The flow through fraction containing [3H]-citrulline was counted for radioactivity using a Beckman 6000 liquid scintillation counter. The NOS activity was expressed as picomoles citrulline per milligram protein per minute. Inhibition of the enzyme was evaluated in all tissues using N-nitro-L-arginine methyl ester hydrochloride (L-NAME—10-6 to 10-4 M). The biochemical characterization of cerebellum NOS showed that spontaneous activity of NOS in the cytosol of hippocampus tissue was greatly reduced when NADPH or Ca2+/ calmodulin was omitted from the incubations medium for the convertion of L-arginine to L-citrulline. The biochemical characterization data of the constitutive NOS isoform in the cerebellum confirmed previous data from our laboratory as Ca2+-independent form of NOS (iNOS activity) represents < 3% [file pone.0125271.s002.tif]
